# The Gap in Electronic Drug Information Resources: A Systematic Review

**DOI:** 10.7759/cureus.2860

**Published:** 2018-06-22

**Authors:** Kerry Anne Rambaran, Hoang A Huynh, Zhen Zhang, Janie Robles

**Affiliations:** 1 Clinical Sciences, Keck Graduate Institute, Claremont, USA; 2 The University of Arkansas for Medical Sciences College of Pharmacy, Arkansas Children’s Hospital, Little Rock, USA; 3 Department of Pharmacy, Memorial Healthcare System, Hollywood, USA; 4 Pharmacy Practice School of Pharmacy, Texas Tech University Health Sciences Center, Lubbock, USA

**Keywords:** drug information, drug information resources, adverse events, medication errors

## Abstract

The landscape of drug information is growing, leading to information overload from various avenues, both scientific and public opinion. The completeness of these resources are not well-studied and no standardizations exist for these databases. Thus, it is not uncommon to have missing information across the drug information resources used by healthcare professionals. Such gaps in these resources may lead to fatal and nonfatal incidences if more than one resource is not consulted. To date, there have been numerous medication errors reported in the literature. In an effort to review the data found in drug information resources, we conducted a comprehensive search of the PubMed, Embase, and EBSCO electronic databases from January 2000 to January 2017, using the terms “drug information,” “medical information,” and “drug information resource.” A total of 14 articles were identified and five were included in our review, which evaluated the differences between drug information resources. Two articles evaluated pharmacogenomics information, one was infectious disease-specific, one evaluated usability as well as other factors, and the last evaluated general content. Overall, there was consistency across the articles in that they each reported on disparities in drug information among several drug information resources. Drug information keeps changing, and it is imperative that healthcare professionals have access to multiple resources to ensure the accuracy and completeness of information. We strongly encourage the standardization of drug information content on drug information resources as well as the information made available from pharmaceutical companies, as it may refine the quality of drug information provided to help prevent medication errors and adverse drug events.

## Introduction and background

Drug information requires thorough evaluation when seeking patient-specific or patient population information. To provide safe and effective care to patients, a fair, balanced, and unbiased approach must be adopted when seeking drug information. As such, the frontline pharmacist and drug information centers are sometimes utilized in addition to electronic drug information resources. However, no single electronic drug information resource is all-encompassing, thus leading to incomplete or inconsistent drug information. An absence of information may lead to an unintended medication error or adverse event. Medication errors are defined as any error occurring in the medication use process [1]. Adverse events are events resulting in unintended harm to the patient by an act of commission or omission rather than by the underlying disease or condition of the patient [2]. Moreover, in the context of regulation and drug safety, adverse events need not have a causal relationship with the therapy or intervention [3]. Adverse drug events are injuries caused by medications and are further classified as medication errors if preventable. The Institute of Medicine (IOM) reported an incidence of approximately 98,000 annual deaths due to medication errors in 1999, which is an underrepresentation of the degree of the problem [4]. Four earlier studies have extrapolated that 140,000-400,000 annual deaths occur, which is four times the IOM-reported estimate [5-9]. Thus, not only are medication errors the most common errors made, but they are also reported to be the third-leading cause of death in the United States (US) [9-10]. Medication labeling is one resource utilized to acquire drug information. Prior to 2006, a medication package insert provided an exorbitant amount of information, resulting in confusion and physician fatigue in searching for answers. In a survey conducted by the Food and Drug Administration (FDA) between 1993 and 1994, it was found that physicians were inundated with information. As a result, physicians requested that labels provide easy access to certain labeling sections as well as a brief synopsis of the most frequently consulted information [11]. Thus, in January 2006, the FDA issued the “Physician Labeling Rule,” which is also known as the “Requirements on Content and Format of Labeling for Human Prescription Drug and Biological Products” [12]. This rule applies to all new drugs approved after December 2000. Additionally, the new labeling requirements (e.g. package insert) now stratify the previous format into three main areas: highlights of prescribing information, full prescribing information (FPI), and limiting the contents of the FPI to half a page [11]. Other resources accessed for drug information include, but are not limited to, drug information handbooks, mobile applications, and databases, of which, most, if not all, of these media obtain information from the package insert. To that end, the package labeling contains only FDA-approved information, not information from the primary literature and, in many cases, post-marketing information. Thus, it is imperative that the resources utilized by providers to obtain drug information are adequate to avoid medication errors as well as adverse drug events. As a result, this review is geared towards identifying gaps in select drug information resources and providing potential solutions to circumvent this issue.

## Review

Methods

We followed the Preferred Reporting Items for Systematic Reviews and Meta-Analysis (PRISMA) guidelines for this systematic review [13]. The objective of this review was to identify all articles published in PubMed, Embase, and EBSCO between January 2000 and January 2017, which assessed drug information resources. Year 2000 was chosen because, based on our research, the first online drug information resource was made available in 2000 [14]. Multiple variations of the following terms were utilized: drug information, medical information, and drug information resource. Additional references were identified from conference proceedings and/or citations in relevant review articles and assessed to find all available papers. We utilized the following criteria for article selection: (1) articles written in English, (2) must be full-text articles, (3) must be a randomized controlled trial, prospective trial, retrospective analysis, case series, exploratory analysis, or cross-sectional study, (4) must include the evaluation of resources from an online resource or smartphone app, which did not include personal digital assistants (PDAs), and (5) must include a comparison of drug information across resources with those speaking solely to the usability of the resources and excluding interaction checker components.

Results

**Figure 1:**
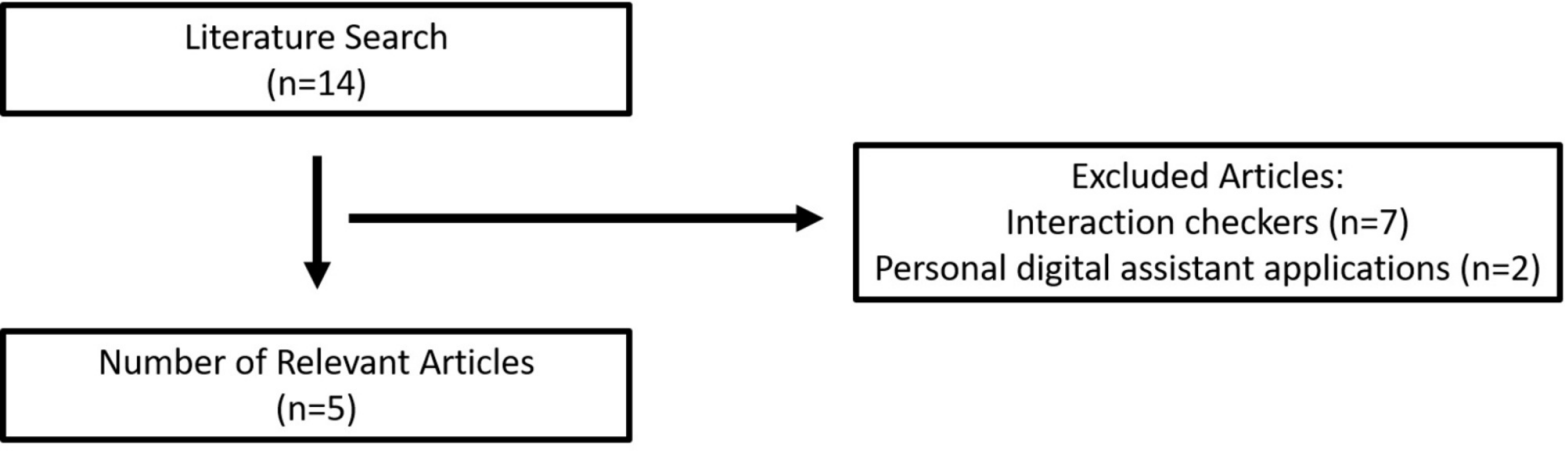
Search flow diagram

In total, the initial search identified 14 articles (Figure 1). After applying the inclusion criteria, seven articles that solely discussed interaction content among various drug information resources and two discussing PDA applications were excluded. Five articles that examined several databases met the inclusion criteria, in which two examined pharmacogenomics information, one examined infectious disease content, one evaluated the scope, ease of use, and comprehensiveness, and one compared the completeness of information when compared to the full prescribing information.

Randhawa and colleagues evaluated drug summaries across Medscape Drug Reference, Lexi-Comp Online, Epocrates Online Premium or Free, Drugs.com, and RxList for accuracy, completeness, and to identify product-specific misinformation. Between August 2014 and January 2015, 270 drug summaries in five drug resources were evaluated. The resources were stratified into healthcare-provider-focused (Medscape Reference, Lexi-Drug, and Epocrates) and consumer-focused (Drugs.com and RxList). The drug summaries were evaluated for any information that was inaccurate, incomplete, or omitted when compared to the full prescribing information, instructions for use, and/or medication guide. For resources that have a smartphone application, the online version was assessed. From the 270 drug summaries evaluated, 782 errors were found with the following breakdown: dosage and administration (n=149), patient education (n=137), and warnings and precautions (n=123). Within the three healthcare-provider-focused resources, 162 drug summaries were evaluated, with a total of 899 errors being identified and the majority of errors occurring in the warnings and precautions, dosage and administration, and patient education sections [15].

Chang et al. evaluated package inserts, Lexi-Comp Online, Micromedex 2.0, and Epocrates, to determine which provided clinically useful pharmacogenomic information as well as incorporated information from the Clinical Pharmacogenomics Implementation Consortium (CPIC) guidelines. The pharmacogenomic content was evaluated based on the mention of the biological effect of a biomarker, population prevalence, testing recommendations, and the interpretation of the test result and was categorized as "complete," "partial," or "not answered." Additionally, they further examined the degree to which the CPIC guidelines were incorporated into the drug information monographs (the CPIC guidelines “are intended to translate laboratory test results into clinically actionable prescribing decisions”). They specified that of the four resources, only Lexi-Comp Online, in a specifically delineated pharmacogenomics section, provided pharmacogenomics information for all 27 drugs with only eight drugs entailing CPIC recommendations, whilst Epocrates provided pertinent information for 13 drugs or less. Notably, only 19% of drug monographs fully incorporated the CPIC recommendations, with only three drugs having full incorporation across all four drug information resources. Lexi-Comp Online provided CPIC recommendations. It was concluded that clinicians should refer to the CPIC guidelines, as most, if not all, of those recommendations are yet to be included in drug information resources [16].

Vaughan and colleagues evaluated whether the pharmacogenomics information contained in the package insert of 65 drugs was also present in Lexi-Comp Online, Facts and Comparisons 4.0, American Society of Health-System Pharmacists' (AHFS), Micromedex 2.0, and Epocrates. The study searched for biomarkers from the FDA package inserts in the five drug information sources aforementioned. Each resource had the opportunity to present biomarker information for 65 drugs, thus a total of 325 opportunities were presented. A subanalysis was performed on the 13 most frequently prescribed drugs in the United States. The reference biomarkers information was reported as the following: Lexi-Comp Online 62/65 times (95.3%), Epocrates Online Free 44/65 times (67.7%), Micromedex 2.0 60/65 times (92.3%), Facts and Comparisons 4.0 50/65 times (76.9%), and AHFS 49/65 times (75.4%). Additionally, two package insert biomarkers (NAT1 and NAT2, 7.7%) were not found in any resource, and 15/26 biomarkers (57.7%) were found in all of the resources. They reported that Lexi-Comp Online provided the most pharmacogenomics information. Moreover, they concluded that clinicians were 6.6 times more likely to miss pharmacogenomics information with Epocrates Free when compared to Lexi-Comp Online [17].

Clauson et al. evaluated Clinical Pharmacology, Micromedex, Lexi-Comp Online, Facts & Comparisons 4.0, Epocrates Online Premium, RxList.com, and Epocrates Online Free according to scope (ability to answer a question), completeness (comprehensiveness), and ease of use. The results, according to scope, were: Clinical Pharmacology 86.7%, Micromedex 83.5%, Lexi-Comp Online 82.9%, Facts & Comparisons 4.0 81%, Epocrates Online Premium 65.2%, RxList.com 65.2%, and Epocrates Online Free 53.2%. With respect to completeness of information, the scores were as follows: Clinical Pharmacology 96.4%, Micromedex 97%, Lexi-Comp Online 95.2%, Facts & Comparisons 4.0 95.8%, Epocrates Online Premium 93.2%, RxList.com 89.6%, and Epocrates Online Free 93.2%. Lastly, with regard to ease of use, the mean number of steps required were as follows: Clinical Pharmacology 3.50, Micromedex 2.70, Lexi-Comp Online 2.16, Facts & Comparisons 3.02, Epocrates Online Premium 1.72, RxList.com 3.17, and Epocrates Online Free 1.66. Composite scores were calculated for each area and Clinical Pharmacology had the highest composite score of 87.1, implying it performed the best. It was further concluded that subscription databases had a statistically wider scope than the free databases studied [18].

The ability of online drug information databases to provide clinical decision support when answering infectious disease-specific queries was evaluated by Polen and colleagues. The subscription drug information databases they reviewed included AHFS Drug Information, Clinical Pharmacology, Epocrates Online Premium, Facts & Comparisons 4.0 Online, Lexi-Comp Online, Lexi-Comp with AHFS, Micromedex, PEPID PDC, and six freely accessible databases including: DailyMed, DIOne, Epocrates Online Free, Internet Drug Index, Johns Hopkins ABX Guide, and Medscape Drug Reference. The eight subscription drug information databases were evaluated for their scope (presence of an answer) and completeness (on a 3-point scale) in answering 147 infectious disease-specific questions. Questions were divided among five classifications: antibacterial, antiviral, antifungal, antiparasitic, and vaccination/immunization. Classifications were further divided into categories (e.g., dosage, administration, emerging resistance, synergy, and spectrum of activity). Scope scores revealed three discrete tiers of database performance: Tier 1 (82%-77%), Tier 2 (73%-65%) and Tier 3 (56%-41%), which were significantly different from each other (p < 0.05). The top-tier performers: Micromedex (82%), Medscape Drug Reference (81%), Lexi Comp with AHFS (81%), AHFS (78%), and Clinical Pharmacology (77%) answered significantly more questions compared to other databases (p < 0.05). The top databases for completeness were: Micromedex (97%), DailyMed (96%), Internet Drug Index (95%), and Medscape Drug Reference (95%). Subscription databases performed better than free databases in all categories (p = 0.03). Databases suffered from 37 erroneous answers for an overall error rate of 1.8% [19].

Discussion

Drug information resources are available through various media and are valuable tools that healthcare providers utilize to find answers on pharmaceutical products. However, the completeness of these resources is yet to be validated and, as a result, poses a risk for product misuse and patient harm.

Although printed package inserts and drug monographs are available for easy access, the most recent interactions and adverse events are found in primary literature and case reports. However, in practice, time constraints may pose a potential barrier in seeking out the most current information. Online resources, such as Lexi-Comp Online, Micromedex, and Epocrates, provide summaries and detailed monographs for drugs, diseases, and other areas and are readily available on mobile applications. In general, the average number of adverse reactions listed for one drug is approximately 500 [20-21]. Listing over 500 adverse drug reactions may result in information overload, and it also has the potential to diminish the importance of precautions and warnings. As a result, resources are constructed with an abridged version of the package insert and some databases may include non-FDA-approved indications, primary literature and post-marketing information from the FDA. However, paraphrasing and summarizing information brings its own inherent risk of misinformation and lack of completeness. This was concluded by Cheng and colleagues in 2011, in which the indexed boxed warning for 71 drugs was compared between Black-BoxRx, DrugDex, Facts and Comparisons, Epocrates, Lexi-Comp, PDR.net, and the product package insert [22]. They reported that resources, which were predisposed to summarizing, paraphrasing, and compressing information, were more likely to contain conflicting information and thus provide room for errors and/or adverse drug events to occur.

The studies included in this review assessed the completeness and content of various drug information resources. Moreover, there is variability in the results obtained across studies. Clauson and colleagues in 2007 reported Clinical Pharmacology as having the highest scores in the areas of scope, completeness, and ease of use when compared to other resources [18].

Of note, the articles included in our review did not speak to the interaction checker component of some drug information resources. In 2015, Roblek and colleagues conducted a systematic review of the literature to compare the most commonly used drug-drug interaction (DDI) databases [23]. The articles included in their review reported on one or more of the following resources: Drug-Reax software from Micromedex, Healthcare Series, Drug Interactions Facts software, Lexi-Interact software, Pharmavista, EpocratesRx, MediQ, and Drug interaction checker. They reported that the most commonly used resource was Drug-Reax from Micromedex due to its high sensitivity, which, in turn, also means it populates numerous interactions, some of which may not be clinically relevant. They further commented that the online version of Lex-Interact is updated daily whilst Pharmavista and Drug Interaction Facts are updated monthly and Drug-Reax from Micromedex is updated every three months. Thus, the frequency with which information is updated plays an important role in alleviating potential interactions.

Another component to consider is the usability of the drug information resources, as this can play a significant role in obtaining pertinent information in a timely manner. In 2010, Mountford and colleagues qualitatively and quantitatively assessed Lexi-Comp, Clinical Pharmacology, and Micromedex (2008) in the areas of quality, performance, and usability utilizing 15 drug information questions covering 17 different categories of drug information [24]. With regards to database quality, Lexi-Comp scored highest when compared to Micromedex and Clinical Pharmacology (2.6 vs. 2.2 vs. 1.6), albeit not statistically significant. In the area of performance, Lexi-Comp scored the highest (2.7), when compared to Clinical Pharmacology (2.4) and Micromedex (2.3). The authors noted that "Lexi-Comp outperformed the other databases in 5 of the 17 drug information categories: drug interaction, monitoring, pharmacology, foreign/Canadian/newly approved drugs, and herbal/non-prescription drugs." As it pertained to database usability, Lexi-Comp scored the highest (4.1) in the satisfaction domain when compared to Clinical Pharmacology (3.6) and Micromedex (3.1). Moreover, Micromedex received the lowest scores in regards to layout, navigation, and speed domains (3.4), when compared to Clinical Pharmacology and Lexi-Comp Online (4.0) (p<0.05). Lastly, they reported that Lexi-Comp was ranked as the most preferred database and Micromedex the least preferred.

Several limitations to our review must be noted. We are aware there are other resources that can be utilized in the quest for information such as point-of-care summaries made available through EBM Guidelines, DynaMed, and other similar resources. We are also aware that e-textbooks made available by DynaMed, UpToDate, and MDConsult may also be used to obtain information. Our review is limited in that articles discussing their evaluations of these resources were not included. Moreover, we chose to exclude articles that evaluated PDAs, as we think mobile applications are becoming more and more available. Of note, the studies included are not without limitations. Each study utilized its own subset of questions to analyze the resources. As such, if a standardized set of questions was utilized, perhaps similar results may be obtained. Additionally, the resources were evaluated in differing years and differing time periods with differing versions of the resource(s). Thus, the frequency at which these resources are updated was not accounted for and may skew the results. Interestingly, the excipients listed in the FDA-approved package labeling may be changed by the manufacturer without being required by the FDA to update the package insert. As such, a telephone call to the manufacturer may be required to provide the assurance of the actual ingredients of a product at any given time, thus making the process of acquiring drug information a more cumbersome one. Despite these limitations, the studies bring to light important points to consider.

Until there is a standardized process for how the contents of drug information resources are selected, the methodology utilized in searching for drug information should be reinforced to address the potential gaps in resources. Furthermore, based on the update timeline provided by Roblek, the delay in providing new information may result in patient harm, albeit unintentional [23]. Thus, it is imperative that healthcare providers utilize multiple drug information resources to “double check” the information found in one resource. This will enable the practitioner to identify any gaps and possibly prevent errors. Of note, this may be a limitation, as healthcare providers may be restricted to the drug information resources purchased by their institution, if provided, or that they may have purchased independently. As previously mentioned above, subscription resources may provide more in-depth information when compared to its free counterpart, which ultimately impacts patient safety and, thus, patient care. In such cases, the package insert(s) should be consulted, as they are inexpensive and, as aforementioned, may contain the most comprehensive FDA-approved information. To our knowledge, there is limited training provided to healthcare professionals, other than pharmacists, in the area of drug information and resource utilization. As such, the inclusion of training in these areas may be of importance to ensure that a healthcare provider can compose a comprehensive response to a drug information question. Additionally, pharmacists are believed to be drug experts, thus consultation with a pharmacist for drug information is a very inexpensive and effective resource.

## Conclusions

There are several issues associated with medication errors, and access to complete drug information is only one contributing factor. The lack of complete and accurate drug information can lead to medication errors resulting in preventable adverse drug events in adults as well as in special populations. Standardizing the content of drug information resources or the process of acquiring drug information can help mitigate medication errors and adverse drug events. In the interim, healthcare providers should utilize all available resources, including pharmacists, as a source of drug information.
